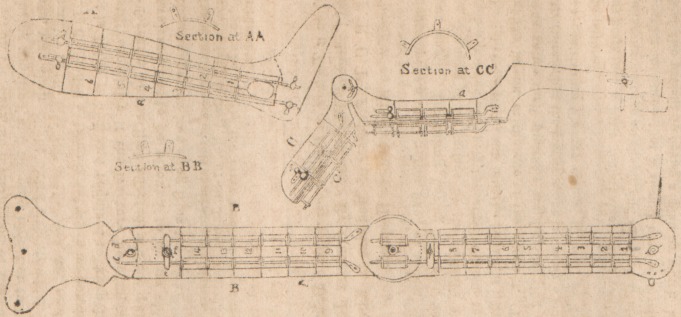# Splint for Compound Fracture

**Published:** 1864-10

**Authors:** S. Stacy Skipton

**Affiliations:** Assistant Surgeon 78th Highlanders.


### CHRONICLE OF MEDICAL SCIENCE.

*Splint for Compound Fractures.* By S. STACY SKIPTON, M. D., Assistant-Surgeon 78th Highlanders.

I am anxious to bring to the notice of the members of the Royal United Service Institution, by means of this paper, a design of an apparatus or splint for "compound" fractures of the limb, *i. e.*, those fractures accompanied with a wound which exposes the fractured bone to the air, such as are caused by gun-shot or other missiles of war.

In the surgical treatment of these injuries, in themselves always serious to the safety of the limb, if not actually dangerous to life, the great object is to apply a splint to support the broken bones in their proper position, at the same time that the wound or wounds in the limb may be left exposed for the application of the requisite dressings.

In the military service, and especially with an army in the field, it cannot be expected that the officers of the medical department can have access to the same resources that are available to the surgeons of a civil hospital, who are surrounded by all the materials which a liberal management permit them to make use of, modify and cut up, if need be, in order to meet the requirements of any one particular case under their care, and so render these materials useless for future casualities, unless they present precisely the same conditions as the former one. With medical officers in the army, on the contrary, economy in the amount of supplies of surgical apparatus is a primary consideration, and especially so under those circumstances in which they are likely to be most required with an army in the field in the presence of the enemy.

In the hospitals at Scutari and in the Crimea in 1854–'55, when the wounded arrived from the battle-fields of Alma, Balaklava, and Inkermann, and from the trenches before Sebastopol, it was distressing to see them lying with fractures of the limbs from gun-shot injuries, for which no apparatus could be extemporized to meet the requirements of the vast majority of these cases. The medical officers were therefore compelled to use the ordinary splints from the stores, and, unfortunately, from the site of the wound being in the course of the splint, and requiring washing and dressing every day, the poor sufferer, officer or private, was daily put to the severe pain consequent upon the splint being removed, the wound cleaned and dressed *without any adequate support to the injured limb*, and the splint itself, which should have rendered this needful support to the broken bones, re-applied. Only those who have themselves suffered from a broken limb, and know what is the pain of having "the bones set" can form an idea of the suffering entailed upon our wounded; and, when we consider the constant state of fever which was maintained by this frequent but unavoidable meddling with the broken bone, it is not to be wondered at that so many of these severe injuries terminated unfavorably, necessitating, at the least, the sacrifice of the limb.

As, then, the requirements of the military service deny to a surgeon in the field the great advantages possessed by a civilian surgeon, and render it necessary for the former to regard, while consistent with their complete efficiency, the portability, simplicity and durability of his surgical apparatus, with economy also in the number and sizes of the splints he carries in his hospital stores, an apparatus or splint so designed as to meet all these reqeirements struck me as being not only desirable, but exceedingly necessary, and such, I trust, will that be found a description of which I have now the gratification of submitting. The shape of the splints for the various limbs remains the same as those in common use, and the material best fitted for rough usage on a campaign, and which is by far the most cleanly of all, is the thin sheet iron laguered over, such as the ordinary splints have heretofore been made of. My object being to provide a splint, the surface of which miget be capable of being interrupted at any part of its length, where, when applied to the limb, it might cover the site of a wound. I have taken away one of the common splints, cut across into transverse strips, each of a width sufficient when removed to uncover a space the size of a gun-shot wound, and at the back of these transverse strips placed vertical plates having holes pierced therein for a longitudinal bar, running along the back of the splint, to pass through; the terminal pieces of the splint are fixed to these longitudinal bars by rivets or screws at either end.

The accompanying drawing of the various splints, depicting a side-plan view of a splint for the arm and forearm, leg and thigh respectively, show each to be composed of transverse strips (see cut *a a*) which, placed side by side, form the shape of the required splint; upon each of these are fixed vertical plates, *b b*, (shown in a front view at the sections A, A, B, B, C, C,) which have a hole pierced therein for a longitudinal bar, *d d* to pass through; each terminal piece of the splint is supplied with pillars *e*, *e*, and set screws *f*, *f*, or rivets to keep these pieces in their respective positions and required distances on the bars. Each transverse strip being numbered consecutively, no mistake need occur by their becoming misplaced, and thereby disarranging the shape of the splint. By loosening the screws and withdrawing the terminal piece from the bars, any one or more of the transverse pieces covering the site of a wound may be removed and set aside, and the terminal piece and the others replaced in their former position. The splint thus applied, and bound on the limb with straps passing under the bars, presents an interruption on its surface, and, by leaving the wound uncovered, gives facility for cleansing and dressing it, without disturbing the support of the splint on the limb. The distance between the bars and the surface of the splint, or rather of the limb to which it is applied, renders any manipulation to the wound practicable and easy, and at the same time the patient is saved the agony and suffering, and consequent irritative fever, caused by having to remove and re-apply the splint to the broken limb after dressing the wound. Another advantage attendant upon this apparatus is, that the "pads" or cushions between the splint and the limb, and the bandages or straps which secure it to the limb, are more easily retained in a cleanly state; a desideratum to be appreciated chiefly by those who have served and suffered these injuries in a hot climate—such as that in which our campaigns have hitherto been carried on.

I have described the principal advantages which this apparatus possesses above the common splint heretofore in use; but there is one other yet to be mentioned, and which, with the above, it exclusively presents; and one especially to be appreciated by our medical officers, whose resources as regards the numbers of the various sizes of splints must be considerably limited, whether at home or abroad. This consists in the same splint which, at the full length of the longitudinal bars, would be sufficiently long for a tall man, being capable of reduction in length by removing one or more of the transverse strips, and bringing down the terminal pieces along the bars, and so making the splint short enough for a drummer boy; thus relieving another great necessity of the service, economy in the numbers of the splints supplied to a force, and ensuring more comfort to the patient than he would have experienced from such apparatus as we had for use at Scutari, in 1854, and later, during the Indian mutiny, where, in many instances, owing to the enormous number of wounded suddenly taken under treatment, the splints were, in addition to their great disadvantages above referred to, either too short or too long for some particular case.

While the above would appear to be sufficient to recommend its adoption in those instances in which there is a wound co-existent with the fracture, this splint may also be used in cases in which there is no wound, in which the fracture is styled "simple." In these the transverse strips would be left on the bars, no interruption of the surface of the splint being required, and the only objection to be raised against them, viz: their slight extra weight, which the additional quantity in the material of the bars, &c., renders unavoidable—is discarded as immaterial when we consider that the patient is lying in bed, and required to keep his limb perfectly still, especially if the fracture be compound. If the arm or forearm be the limb affected, and the patient be so far convalescent as to be allowed to be up, this extra weight is then supported in a sling from the neck.

NOTE.—The manufacturers of this apparatus are Messrs. Whicker & Blaise (late Savigny & Co.,) No. 67 St. James' street, Pall Mall, S. W., (London) who supply the army medical department with surgical instruments.

## Figures and Tables

**Figure f1:**